# Sleep-related painful erection, erectile dysfunction and psychological distress

**DOI:** 10.3389/fpsyt.2026.1778388

**Published:** 2026-03-20

**Authors:** Ludek Fiala, Jiří Lenz

**Affiliations:** 1 Faculty of Medicine in Pilsen, Charles University, Pilsen, Czechia; 2 First Faculty of Medicine, Charles University, Prague, Czechia; 3 General University Hospital in Prague, Prague, Czechia; 4 University Hospital Pilsen, Pilsen, Czechia; 5 nojmo Hospital, Znojmo, Czechia; 6 University of Veterinary and Pharmaceutical Sciences Brno, Brno, Czechia

**Keywords:** erectile dysfunction, hyperprolactinemia, parasomnia, sleep-related painful erections, stress response

## Abstract

**Background:**

Sleep-related painful erections (SRPE) are a rare parasomnia characterized by recurrent nocturnal penile pain occurring predominantly during rapid eye movement (REM) sleep, while erections during wakefulness remain painless. SRPE may lead to sleep disturbance, sexual avoidance, and psychological distress. Although its pathophysiology remains unclear, psychoneuroendocrine mechanisms have been proposed.

**Methods:**

In this exploratory case–control study, fourteen men with SRPE were compared with fourteen men diagnosed with psychogenic erectile dysfunction without a history of SRPE. All participants underwent polysomnography, clinical and urological examination, hormonal assessment, and psychometric evaluation using the International Index of Erectile Function-5 (IIEF-5) and the Trauma Symptom Checklist-40 (TSC-40). Hormonal parameters were obtained from fasting morning blood samples. Non-parametric statistical methods were applied.

**Results:**

Men with SRPE showed significantly lower erectile function and greater psychological distress compared with controls (p < 0.05). Mean prolactin levels were higher in the SRPE group but did not differ significantly at the group level (p = 0.089). Within the SRPE group, prolactin levels were negatively correlated with erectile function and positively correlated with psychological distress (nominal p < 0.05), whereas no such associations were observed in the control group.

**Conclusion:**

SRPE may be associated with a specific psychoneuroendocrine profile in which psychological distress and prolactin dynamics are linked to secondary erectile dysfunction. These exploratory findings support a multidimensional clinical perspective integrating sleep-related, psychological, and neuroendocrine factors and warrant further investigation in longitudinal studies.

## 1 Introduction

Sleep-related painful erections (SRPE) represent a rare and underrecognized parasomnia characterized by recurrent nocturnal penile tumescence accompanied by pain, typically occurring during rapid eye movement (REM) sleep, while erections during wakefulness remain painless and physiologically intact (1). Although SRPE has been reported sporadically in the literature for more than three decades, its underlying mechanisms remain insufficiently understood, and the condition is frequently misdiagnosed or approached primarily from a urological perspective.

Emerging clinical observations suggest that SRPE may involve a complex interplay of sleep physiology, autonomic regulation, pain processing, and neuroendocrine factors. Rather than representing a purely peripheral or urological phenomenon, SRPE may be associated with central mechanisms related to REM sleep instability, heightened sympathetic activity, altered nociceptive processing, and stress-related hormonal modulation.

Clinically, SRPE is associated with significant sleep fragmentation, fatigue, anxiety, and avoidance behaviors related to both sleep and sexual activity. Many patients report impaired erectile function, which may develop in the context of anticipatory anxiety, conditioned fear responses related to nocturnal pain, heightened autonomic arousal, and relational stress. Unlike organic erectile dysfunction associated with vascular or metabolic conditions, erectile difficulties observed in SRPE are frequently reported in psychologically vulnerable individuals with increased stress responsivit (2).

Previous case reports and small observational studies have described potential associations between SRPE, alterations in prolactin levels, and increased sympathetic nervous system activity during REM sleep, raising the hypothesis that stress-related neuroendocrine processes may play a role in symptom expression (3).

Prolactin is a pituitary hormone known to influence sexual behavior, libido, and erectile function and has been discussed as a potential marker of psychological distress and stress-related sexual dysfunction. Elevated prolactin levels have been associated with reduced sexual desire, erectile impairment, and alterations in central dopaminergic tone, although findings across clinical populations remain heterogeneous.

Experimental and translational studies suggest that acute psychological stress may influence REM sleep architecture and modulate prolactin secretion in both human and animal models. Prolactin release is known to interact bidirectionally with dopaminergic and serotonergic systems, which are involved in the regulation of REM sleep, pain perception, and sexual arousal. These interactions provide a theoretical framework for considering SRPE as a condition potentially linking stress-related neuroendocrine responses with REM sleep–dependent symptom expression.

Despite these conceptual considerations, there remains a significant gap in the clinical literature. To date, no controlled clinical studies have systematically examined the relationships between prolactin levels, psychological distress, and erectile function in men with SRPE using standardized psychometric assessment and hormonal profiling. Existing reports are largely limited to case studies or small descriptive series without integrated psychometric or neuroendocrine analyses.

### 1.1 Aim of the study

The present exploratory case–control study addresses this gap by examining hormonal and psychological correlates of erectile function in men with SRPE compared with men diagnosed with psychogenic erectile dysfunction without painful nocturnal erections.

### 1.2 Hypothesis

We hypothesized that, in men with SRPE, serum prolactin levels would be positively associated with psychological distress and negatively associated with erectile function, and that these associations would not be present in men with psychogenic erectile dysfunction without SRPE (4). By characterizing these associations, the study aims to contribute to a more refined understanding of the potential psychoneuroendocrine profile of SRPE and to inform future multidisciplinary research approaches.

## 2 Methods

### 2.1 Study design and setting

Given the rarity of sleep-related painful erections (SRPE), this study was designed as a prospective exploratory case–control investigation conducted between January 2020 and June 2023 at two academic clinical centers specializing in sexual medicine and sleep disorders in the Czech Republic. The study was approved by the institutional ethics committee (Approval No. 226/20) and conducted in accordance with the Declaration of Helsinki. All participants provided written informed consent.

### 2.2 Participants and diagnostic criteria

A total of 28 adult men were enrolled and divided into two groups.

SRPE group (n = 14): Patients diagnosed with sleep-related painful erections based on polysomnographic evidence of nocturnal penile tumescence accompanied by pain.

ED control group (n = 14): Patients diagnosed with psychogenic erectile dysfunction without any history of nocturnal painful erections or SRPE symptoms.

All participants in the ED control group underwent comprehensive multidisciplinary diagnostic evaluation. Organic etiologies of erectile dysfunction were systematically excluded, including structural urological pathology (including prostate disease), neurological disorders or post-traumatic conditions, vascular erectile dysfunction, cardiovascular disease or hypertension, endocrine disorders, and relevant pharmacological influences. The rationale for selecting a psychogenic ED control group was to ensure comparability with SRPE patients in terms of psychological stress exposure, while lacking the defining clinical feature of SRPE.

Inclusion criteria: male sex, age ≥ 18 years, stable heterosexual partnership (≥ 6 months). For the SRPE group: at least three episodes of painful nocturnal erections per week for ≥ 1 month, confirmed by polysomnography. For the ED control group: clinically significant psychogenic erectile dysfunction (IIEF-5 ≤ 21) without SRPE.

Exclusion criteria included endocrine disorders, neurological or major psychiatric conditions, use of psychotropic or hormonal medication, structural urological abnormalities, history of pelvic trauma, moderate-to-severe obstructive sleep apnea, and clinically relevant vascular conditions, including known or treated arterial hypertension. These exclusion criteria were applied to both groups to minimize potential confounding organic, neurological, endocrine, and vascular factors.

### 2.3 Sleep and clinical assessment

All participants underwent overnight video-polysomnography using standardized criteria of the American Academy of Sleep Medicine. Nocturnal penile tumescence and rigidity were recorded using RigiScan™. Pain temporally associated with REM-related erections was documented by patient-reported awakening and corroborated by NPTR recordings (5).

### 2.4 Hormonal evaluation

Fasting venous blood samples were obtained between 08:00 and 09:00 a.m. Hormonal parameters included prolactin, total and free testosterone, LH, SHBG, DHEA-S, TSH, and PSA. Hormonal measurements were conducted blinded to group allocation. Laboratory personnel were not informed of participants’ clinical status.

### 2.5 Psychometric assessment

Erectile function was assessed using the International Index of Erectile Function-5 (IIEF-5) (6). Psychological distress was assessed using the Trauma Symptom Checklist-40 (TSC-40), which measures symptom dimensions including anxiety, sleep disturbance, dissociation, sexual discomfort, and affective distress (7).

In the present study, the TSC-40 was used to quantify stress-related psychological symptom burden rather than to diagnose trauma exposure per se.

### 2.6 Statistical analysis and data

Data distribution was assessed using the Shapiro–Wilk test. As several key variables deviated from normality, non-parametric statistical methods were applied throughout the analysis.

Between-group comparisons were performed using the Mann–Whitney U test. Associations between hormonal parameters, erectile function, and psychological distress were examined using Spearman’s rank correlation coefficient (ρ). Given the exploratory nature of the study and the limited sample size, correlation analyses were interpreted as hypothesis-generating. To reduce the risk of type I error, p-values were adjusted using the Holm–Bonferroni method, with both unadjusted and adjusted values reported. For key associations, 95% confidence intervals were estimated using bootstrap resampling. A *post hoc* power analysis was conducted to provide contextual information regarding sensitivity to detect moderate-to-strong associations and should be interpreted cautiously.

Data are presented descriptively as means with standard deviations or medians with interquartile ranges, as appropriate. Data visualization was used to illustrate group-level differences and associations and included bar charts, boxplots, and scatter plots. All statistical analyses were performed using STATISTICA software, version 12 (TIBCO Software Inc.).

## 3 Results

### 3.1 Participant characteristics

A total of 28 men were included in the final analysis, comprising 14 patients with SRPE and 14 patients with psychogenic erectile dysfunction without SRPE. The two groups were comparable with respect to age, body mass index, and baseline metabolic parameters (all p > 0.05), indicating adequate matching and minimizing major confounding demographic effects.

Participants in the SRPE group reported a mean of 4.2 ± 1.1 episodes of painful nocturnal erections per week, with a mean self-reported pain intensity of 6.8 ± 1.4 on a 0–10 numeric rating scale. No participants in the control group reported pain during nocturnal erections.

### 3.2 Erectile function and psychometric outcomes

The SRPE group demonstrated significantly lower erectile function compared to controls, as measured by the IIEF-5 (median [IQR]: 12.0 [10–14] vs. 14.0 [12–17]; p < 0.05).

Psychological distress, assessed using the TSC-40, was significantly higher in the SRPE group than in the control group (median [IQR]: 48.5 [41–53] vs. 42.4 [36–47]; p < 0.05), indicating a greater burden of anxiety-, sleep-, and distress-related symptoms.

**Figure 1:**
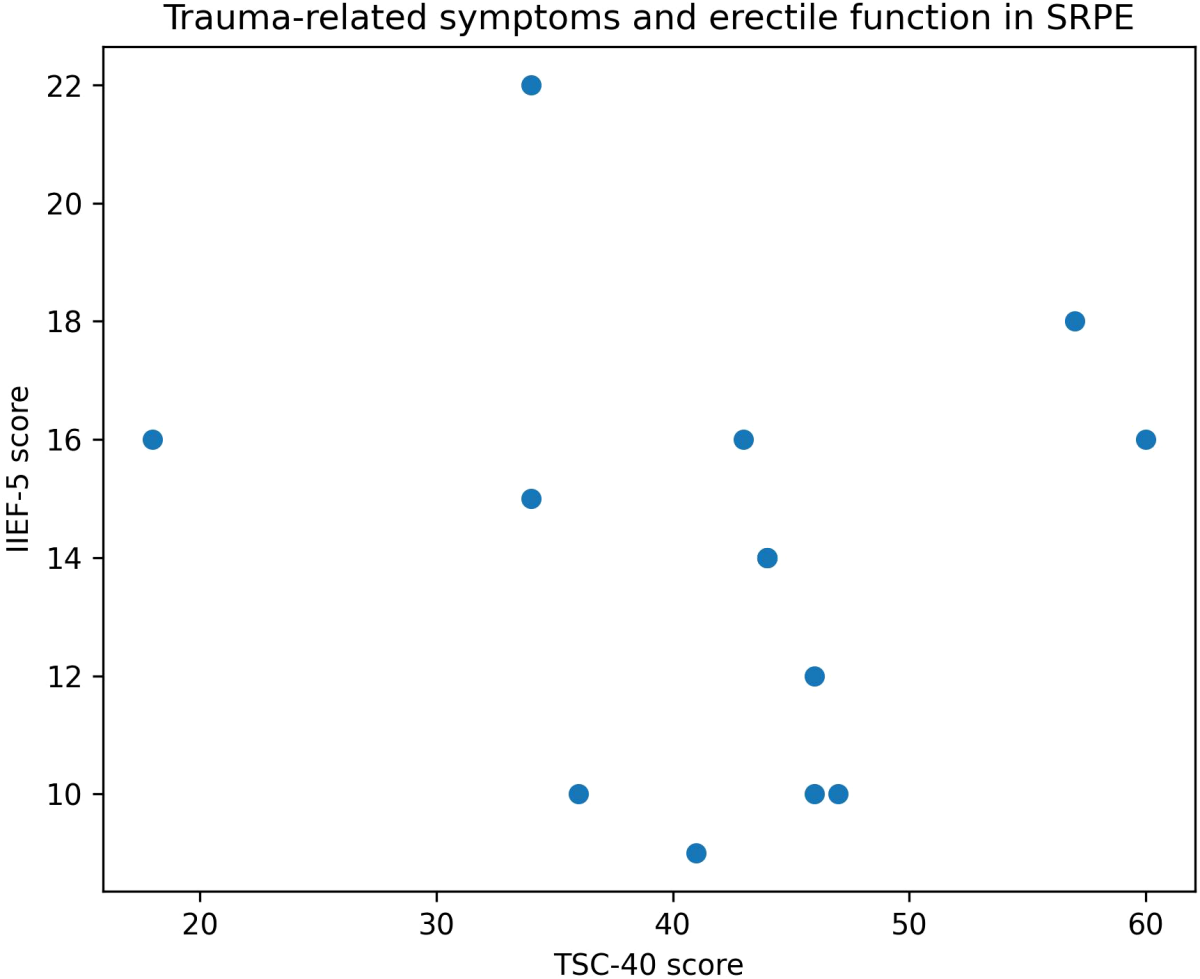
Trauma‐related symptoms and erectile function in SRPE.

Group differences in erectile function and psychological distress are illustrated in **Figure 1**.

### 3.3 Hormonal findings

Mean serum prolactin levels were higher in the SRPE group compared with controls (15.23 ± 5.81 µg/L vs. 10.94 ± 5.92 µg/L); however, this between-group difference did not reach statistical significance (p = 0.089).

No significant between-group differences were observed in free testosterone, LH, SHBG, DHEA-S, TSH, or PSA levels (all p > 0.05).

### 3.4 Correlation analyses

Correlation analyses revealed associations exclusively within the SRPE group. In this group, serum prolactin levels were negatively correlated with erectile function as measured by the IIEF-5 and positively correlated with psychological distress as measured by the TSC-40 (nominal p < 0.05).

A weak, non-significant positive association between free testosterone levels and erectile function was observed in the SRPE group. No significant correlations between hormonal parameters, erectile function, and psychological distress were observed in the control group (all p > 0.05).

### 3.5 Summary of key findings

In summary, men with SRPE exhibited greater psychological distress and poorer erectile function compared with men with psychogenic erectile dysfunction without SRPE. While between-group differences in prolactin levels did not reach statistical significance, significant associations between prolactin, psychological distress, and erectile function were observed within the SRPE group only (8).

**Figure 2:**
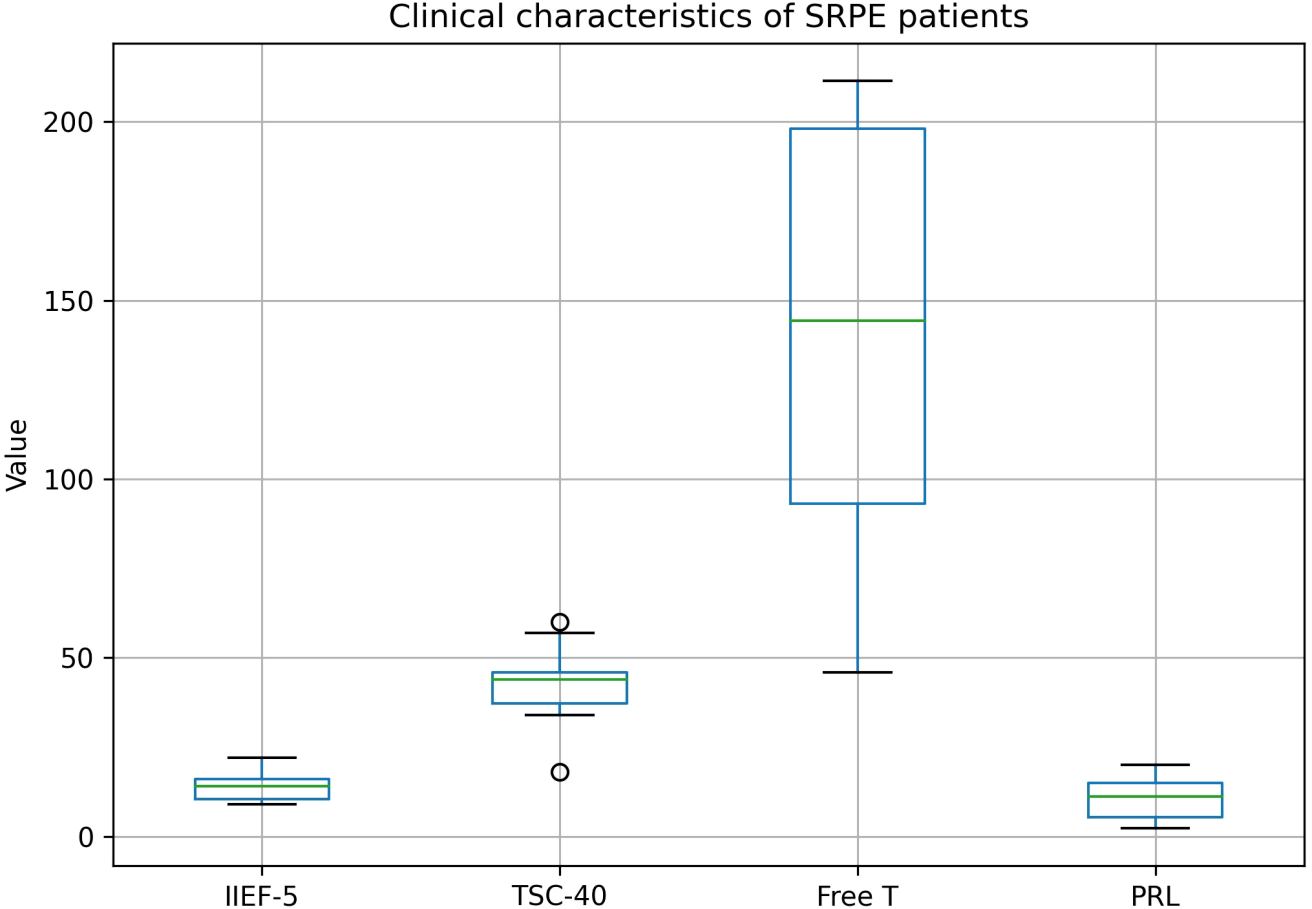
Clinical characteristics of SRPE patients.

**Figure 3:**
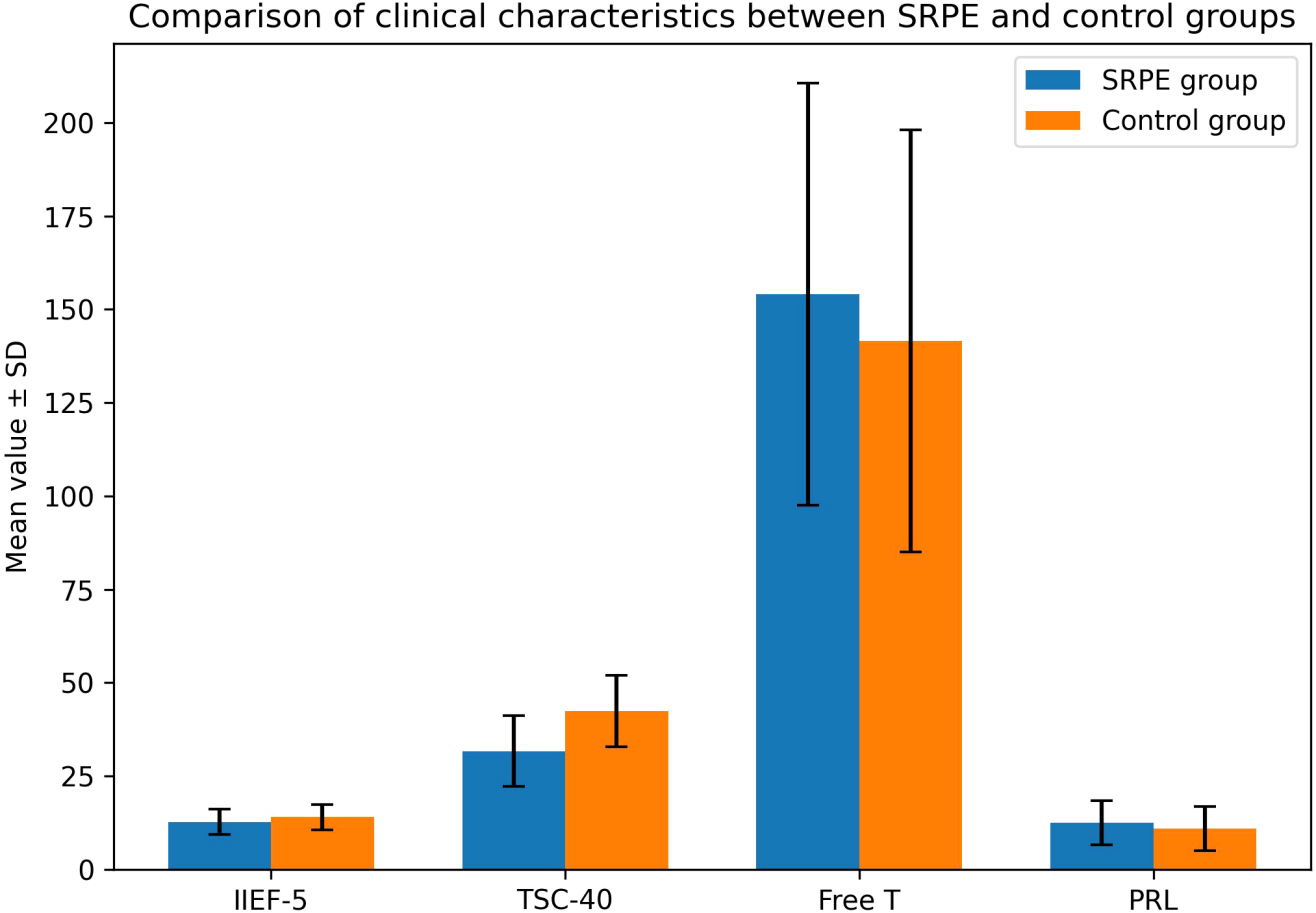
Comparison of clinical characteristic s between SRPE and control groups.

These findings suggest that interactions between psychological distress, neuroendocrine factors, and erectile function (9)may be particularly relevant in SRPE and warrant further investigation in larger and longitudinal studies (10) (**Figures 2**, **3**).

## 4 Discussion

### 4.1 SRPE as a psychoneuroendocrine condition

The present study provides exploratory evidence suggesting that sleep-related painful erections (SRPE) may be associated with a specific psychoneuroendocrine profile involving psychological distress, prolactin dynamics, and erectile dysfunction. Unlike men with psychogenic erectile dysfunction without SRPE, patients with SRPE demonstrated significant within-group associations between prolactin levels, psychological distress, and erectile function.

These associations were not observed in the control group, indicating that the interaction between psychological and neuroendocrine factors may be particularly relevant in SRPE. However, given the cross-sectional design and limited sample size, these findings should be interpreted as hypothesis-generating rather than confirmatory.

### 4.2 Role of REM sleep and stress reactivity

REM sleep is characterized by physiological autonomic variability, including fluctuations in parasympathetic and sympathetic activity. In susceptible individuals, such autonomic changes may be temporally associated with increased pain perception and neuroendocrine responses (11).

Previous experimental and translational studies suggest that psychological stress can influence REM sleep architecture and modulate prolactin secretion. In this context, SRPE may represent a clinical condition in which REM sleep–related autonomic changes interact with stress-related neuroendocrine mechanisms, although the precise directionality and causality of these processes cannot be determined from the present data.

### 4.3 Erectile dysfunction as a secondary phenomenon

Erectile dysfunction observed in patients with SRPE may develop in the context of multiple interacting factors, including anticipatory anxiety related to nocturnal pain, conditioned fear responses, sleep fragmentation, and psychological distress. Stress-related neuroendocrine changes, including alterations in prolactin levels, may further contribute to impaired erectile function (12).

Importantly, the present findings do not allow determination of whether psychological distress and prolactin alterations are primary contributors to SRPE, secondary consequences of recurrent nocturnal pain, or part of a bidirectional process.

### 4.4 Clinical implications

Although the present study is exploratory, the observed associations suggest that SRPE may benefit from a multidimensional clinical perspective that considers sleep-related symptoms, psychological distress, and neuroendocrine factors.

Previous reports indicate that treatment of SRPE is often challenging and may require combined therapeutic approaches, including pharmacological interventions, sleep-focused strategies, and psychological support (13). However, the current study did not evaluate treatment effects, and clinical implications should therefore be interpreted cautiously (14).

### 4.5 Strengths and limitations

This study has several strengths, including the use of standardized polysomnography with NPTR monitoring, validated psychometric instruments, fasting hormonal assessment, and a carefully characterized psychogenic ED control group. To our knowledge, this is one of the first controlled clinical studies to systematically examine psychological and neuroendocrine correlates of SRPE.

Several limitations must be acknowledged. First, the sample size was small, reflecting the rarity of SRPE, which limits statistical power and generalizability. Second, the cross-sectional design precludes causal inference regarding the directionality of associations between psychological distress, prolactin levels, and erectile dysfunction. Third, the study did not include a healthy control group, limiting specificity. Fourth, longitudinal hormonal measurements and additional stress biomarkers (e.g., cortisol or ACTH) were not assessed. Finally, although psychological distress was measured comprehensively, subclinical anxiety and depressive symptoms could not be fully disentangled analytically (15).

### 4.6 Future directions

Future research should focus on longitudinal and multicenter studies to clarify the temporal relationships between psychological distress, neuroendocrine changes, and SRPE symptoms. Repeated hormonal measurements across sleep–wake cycles, inclusion of additional stress-related biomarkers, and integration of neuroimaging approaches may help elucidate underlying mechanisms. Controlled intervention studies are also needed to determine whether targeting psychological distress or neuroendocrine pathways can improve clinical outcomes in SRPE.

## 5 Conclusions

This exploratory case–control study suggests that sleep-related painful erections may be associated with a distinct psychoneuroendocrine profile in which psychological distress and prolactin dynamics are linked to secondary erectile dysfunction. Importantly, these associations were observed exclusively in men with SRPE and were not present in a psychogenic erectile dysfunction control group, supporting the notion that SRPE represents a clinically specific condition rather than a variant of psychogenic ED (16).

Although causal relationships cannot be inferred, the findings highlight the relevance of integrating sleep-related symptoms, psychological distress, and neuroendocrine factors in the clinical assessment of SRPE. From a clinical perspective, SRPE may therefore benefit from a multidisciplinary approach extending beyond purely urological evaluation.

Given the rarity of SRPE and the exploratory nature of this study, the results should be interpreted cautiously. Nevertheless, they provide a rationale for future longitudinal and mechanistic studies aimed at clarifying temporal relationships and identifying potential therapeutic targets.

## Data Availability

The original contributions presented in the study are included in the article/supplementary material. Further inquiries can be directed to the corresponding author.
